# Animal-to-Human Transmission of *Salmonella* Typhimurium DT104A Variant

**DOI:** 10.3201/eid1012.040286

**Published:** 2004-12

**Authors:** Susan W.M. Hendriksen, Karin Orsel, Jaap A. Wagenaar, Angelika Miko, Engeline van Duijkeren

**Affiliations:** * Utrecht University, Utrecht, the Netherlands;; † Federal Institute for Risk Assessment, Berlin, Germany

**Keywords:** Salmonella infections, animal, Zoonosis, Transmission, dispatch

## Abstract

*Salmonella enterica* serovar Typhimurium was isolated from a pig, a calf, and a child on a farm in the Netherlands. The isolates were indistinguishable by phenotyping and genotyping methods, which suggests nonfoodborne animal-to-animal and animal-to-human transmission. Persons in close contact with farm animals should be aware of this risk.

Salmonellae are widespread in humans and animals worldwide. In industrialized countries, nontyphoid salmonellae are an important cause of bacterial gastroenteritis. In the Netherlands, the estimated incidence of salmonellosis is 3 cases per 1,000 inhabitants per year (*1*). In the United States, *Salmonella* is estimated to cause 1.4 million illnesses and 600 deaths annually (*2*). *Salmonella enterica* subspecies enterica serovar Typhimurium can cause infections in humans and animals. Most human cases are foodborne; however, nonfoodborne *Salmonella* infection may be transmitted during contact with animals, contaminated water, or the environment (*3**–**9*). We report apparent transmission of *S*. Typhimurium on a farm.

## The Case

A farm in IJsselstein, the Netherlands, housed 80 dairy cows and 250 finishing pigs (for fattening) in separate sheds. The farmer took care of animals in different stables without changing clothes, and his children had access to all the stables.

In January 2001, the farmer consulted a veterinarian of the Pig Health Unit of Utrecht University regarding a problem with his pigs. In a compartment where 95 pigs (≈6 months of age) were housed, 1 of the pigs was very listless, had a rectal temperature of 41.2°C, and had yellowish diarrhea. Another pig had died suddenly that morning. At that time, the other pigs in the compartment were asymptomatic, but the farmer had noticed diarrhea in several pens a few days earlier. A fecal sample was taken from the ill pig for bacteriologic examination. Despite therapy with enrofloxacin, the pig died. Veterinarians of the Ruminant Department of Utrecht University were consulted 20 days later regarding five 3-week-old calves on the same farm. The calves had diarrhea and fever, and two of them had symptoms of pneumonia. A fecal sample was taken from one of the calves, and the calves were medicated intramuscularly with trimethoprim/sulfadiazine and polymyxin orally. All calves recovered after treatment.

Three weeks after the first veterinarian's visit, the farmer's 5-year-old son became ill with diarrhea and a fever. At that time, the pig was known to have had salmonellosis. Amoxicillin was prescribed for the boy, and a fecal sample was taken for bacteriologic examination. The farmer, his wife, and the other children were not tested because they were healthy.

All three samples (two from animals, one from the child) yielded *Salmonella* after direct plating without pre-enrichment (*10*). No other pathogens were found. Susceptibilities to 17 antimicrobial agents (Table) were assessed by using the National Committee for Clinical Laboratory Standards (NCCLS) broth microdilution method (*11*). Breakpoints given by NCCLS and the Danish Integrated Antimicrobial Resistance Monitoring and Research Programme (*11**,**12*) were used.

**Table:** Three *Salmonella enterica* serovar Typhimurium DT 104A isolates from a boy, a pig, and a calf on a Dutch farm, with MIC values for antimicrobial drugs

Antimicrobial drug	MIC (μg/mL)^a^		
	Porcine strain	Bovine strain	Human strain
Tetracycline	>32 R	>32 R	>32 R
Sulfamethoxazole	>512 R	>512 R	>512 R
Spectinomycin	32 S	32 S	32 S
Chloramphenicol	8 S	8 S	8 S
Florfenicol	8 S	8 S	8 S
Streptomycin	8 S	32 R	16 I
Ampicillin	2 S	2 S	2 S
Neomycin	<2 S	<2 S	<2 S
Amoxycillin-clavulanic acid	<2/1 S	<2/1 S	<2/1 S
Nalidixic acid	<4 S	8 S	<4 S
Gentamicin	<1 S	<1 S	<1 S
Trimethoprim	>32 R	>32 R	>32 R
Colistin sulfate	<4 S	<4 S	<4 S
Trimethoprim-sulfamethoxazole	>8/152 R	>8/152 R	>8/152 R
Ciprofloxacin	<0.03 S	<0.03 S	<0.03 S
Ceftiofur	1 S	1 S	1 S
Kanamycin	<4 S	8 S	<4 S

^a^The categories susceptible (S), intermediate (I), or resistant (R) were assigned on the basis of breakpoints recommended by the National Committee for Clinical Laboratory Standards and the Danish Integrated Antimicrobial Resistance Monitoring and Research Programme (11,12).

Additionally, serotyping based on O and H antigens, according to the Kauffmann-White scheme (*13*); phage typing in accordance with the methods of the Health Protection Agency, London (*14* and L.R. Ward, pers. comm.); plasmid profiling (*15*); and pulsed-field gel electrophoresis (PFGE) after digestion with *Xba*I and *Spe*I (*16*) were performed.

Serotyping and phage typing all three samples identified *S*. Typhimurium DT104A variant, a subtype of DT104 that is similar but not identical to DT104A. Antimicrobial-drug susceptibility tests showed that the salmonellae had identical resistance patterns. They were sensitive to most of the antimicrobial agents tested, except for tetracycline, sulfamethoxazole, trimethoprim, and trimethoprim-sulfamethoxazole; MIC values for these agents were similar (Table). All isolates possessed a single plasmid of ≈7 MDa, and all isolates had the same PGFE pattern after digestion with each of the enzymes (Figure).

**Figure:**
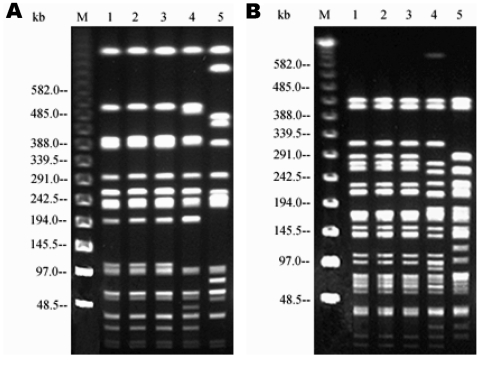
Pulsed-field gel electrophoresis profiles of the three strains after digestion with *Xba*I (A) and *Spe*I (B). Lane M, molecular size markers. Lane 1, porcine strain. Lane 2, bovine strain. Lane 3, human strain. Lane 4, comparison strain *Salmonella enterica* serovar Typhimurium DT104A with a different resistance pattern. Lane 5, *S*. Typhimurium DT104L strain with the common pentaresistance pattern.

## Conclusions

*S*. Typhimurium DT104A variant was isolated from a diseased pig, calf, and child on a Dutch farm. All three strains were typed by phenotypic and genotypic methods and appeared to be identical, which suggests an epidemiologic link. *S*. Typhimurium DT104A isolates are uncommon and show less resistance determinants in comparison to other DT104 isolates (*17**,**18*). The *S*. Typhimurium DT104A variant strain in the present study was resistant to sulfonamides, tetracycline, and trimethoprim-sulfamethoxazole, which is a common resistance pattern of DT104A isolates. Unlike other *S*. Typhimurium DT104 isolates, resistances to ampicillin, chloramphenicol, and florfenicol are rare in *S*. Typhimurium DT104A, as is resistance to kanamycin, neomycin, and gentamicin (*18*).

Because the boy had free access to the stables, we assume that he was infected by direct or indirect contact with animals. The boy was not likely to have been infected with this particular DT104A variant by any other route because this is an uncommon phage type. Transmission of *Salmonella* spp. by direct contact with animals has been reported before (*3**–**9*). Close contact with farm animals is a risk factor for *S*. Typhimurium DT104 infections (*8**,**9*).

The primary source for human disease was difficult to identify, but it was most likely the pigs. Calves were 1 day old when the pig died and 3 weeks old when they became ill, and *Salmonellae* may have been transmitted from pigs to calves shortly after the calves were born. However, the incubation period of salmonellosis is short (1–3 days), and therefore the calves were probably infected when they were nearly 3 weeks old. The farmer, other members of the family, or visitors may have transmitted contaminated pig feces to the calves on dirty boots, clothes, or fomites. The pigs or the calves could have infected the boy. The calves are more likely because the boy's rabbits were housed in the calves' stable and therefore he had more intensive contact with the calves than with the pigs. Another possibility is that the farmer transmitted the infection to the boy as a result of inadequate handwashing, wearing inadequately disinfected footwear, or wearing working clothes indoors.

We advise those who are at high risk for *Salmonella* infection, e.g., farmers, veterinarians, and slaughterhouse workers, to follow general hygiene guidelines. The amount of bacteria shed by hosts is probably much larger in clinical salmonellosis than in the carrier state, and great care must be taken to clean and disinfect hands and tools to prevent spread of the bacteria after contact with clinically ill animals. Veterinarians must inform animal caretakers about the zoonotic aspects of disease when they diagnose a *Salmonella* infection.
